# Genome-Wide Identification and Characterization of *Receptor-Like Protein Kinase 1 (RPK1)* Gene Family in *Triticum aestivum* Under Drought Stress

**DOI:** 10.3389/fgene.2022.912251

**Published:** 2022-07-04

**Authors:** Amna Abdul Rahim, Muhammad Uzair, Nazia Rehman, Obaid Ur Rehman, Nageen Zahra, Muhammad Ramzan Khan

**Affiliations:** ^1^ National Centre for Bioinformatics (NCB), Quaid-e-Azam University, Islamabad, Pakistan; ^2^ National Institute for Genomics and Advanced Biotechnology (NIGAB), National Agricultural Research Centre (NARC), Islamabad, Pakistan

**Keywords:** receptor-like protein kinase 1 (RPK1), abiotic stress, genome-wide studies, phylogenetic relationship, expression patterns, *Triticum aestivum*

## Abstract

*Receptor-like protein kinase* *1 (RPK1)* genes play crucial roles in plant growth and development processes, root architecture, and abiotic stress regulation. A comprehensive study of the *RPK1* gene family has not been reported in bread wheat (*Triticum aestivum*). Here, we reported the genome-wide identification, characterization, and expression patterns of the *RPK1* gene family in wheat. Results confirmed 15 *TaRPK1* genes, classified mainly into three sub-clades based on a phylogenetic tree. The *TaRPK1* genes were mapped on chromosomes 1–3 in the respective A, B, and D genomes. Gene structure, motif conservation, collinearity prediction, and synteny analysis were carried out systematically. A Gene ontology study revealed that *TaRPK1* genes play a vital role during molecular and biological processes. We also identified 18 putative miRNAs targeting *TaRPK1* genes, suggesting their roles in growth, development, and stress responses. *Cis*-Regulatory elements interpreted the presence of light-related elements, hormone responsiveness, and abiotic stress-related motifs in the promoter regions. The SWISS_MODEL predicted the successful models of TaRPK1 proteins with at least 30% identity to the template, a widely accepted threshold for successful modeling. *In silico* expression analysis in different tissues and stages suggested that *TaRPK1* genes exhibited the highest expression in root tissues. Moreover, qRT-PCR further validated the higher expression of *TaRPK1* genes in roots of drought-tolerant varieties compared to the drought-susceptible variety. Collectively, the present study renders valuable information on the functioning of *TaRPK1* genes in wheat that will be useful in further functional validation of these genes in future studies.

## Introduction

Globally, wheat is a staple food and a source of nutrition. In the last 2 decades, the production of wheat increased by up to 1% annually (Manès et al., 2012), but this increase is not enough to meet the demand of the population, which will increased from 7.8 billion to 9.7 billion in 2050 (Roser and Ortiz-Ospina, 2013). The climatic changes including abiotic and biotic stresses are the main causes which extremely effect the quality and yield of the crops. To face these challenges, it is vital to explore the crop genotypes that can stand up to all of these hurdles. Plants are immobile in nature; they don’t move here and there in search of food, but their roots do. The root is the major organ that has a crucial role in the adaptation of the plant to its unfavorable environment. Root systems captivate the water and nutrients essential for the growth and maintenance of plant (Alahmad et al., 2019; Grzesiak et al., 2019). Hence, improved root system overcomes the challenges of the harsh environment and might enhance crop production (Djanaguiraman et al., 2019; Danakumara et al., 2021; Rasool et al., 2021).

Drought is one of the major abiotic stresses caused due to scarce rainfall that affects productivity. An increase in drought in the coming 30 years will have adverse effects on crop yield with 6–12 bushels/acre (Zargar et al., 2017). Creation of drought tolerance is a very complicated because many genes such as *TaER1, 2,* and *3, TaZFP34, TaWRKY1, 10, 33, 44*, and *93*, *TaDR O 1*, and *TaRAP2.1* directly or indirectly involved. In animals, *receptor protein kinases (RPKs)* are the genes which play a significant role in the stimulation of hormones and other growth factors (Fantl et al., 1993). In plants, similar to animals’ *RPKs,* there is a *receptor-like protein kinase (RLK)* gene family. The *RLK* family is a huge family of genes found in many plants. The typical *RLK* structure comprises an extracellular domain at the N-terminal, a membrane helix, and an intracellular conserved kinase domain (KD) at the C-terminal. The extracellular domains of the *RLK* family are highly diverged, which results in the differentiation of *RLKs* into 17 distinct subfamilies, including the *receptor-like kinases* (Mishra et al., 2021). The *LRRKs* (*leucine-rich repeat kinase*) represents biggest subfamily of *RLK* with 531 *TaLRRK* genes in wheat (Sharma et al., 2016), comprising of ECD (extracellular domain) to receive signals, TM (transmembrane) region to bound it to cell membrane and cytoplasmic kinase domain for phosphorylation of substrate (Gou et al., 2010; Dievart et al., 2020). The *LRRKs* has numerous roles in plants as it is involved in initiating innate defense at front-line against microbial pathogens (Nejat and Mantri, 2017), morphogenesis, organogenesis, hormone signaling, abiotic, and biotic stress regulation in plants (Diévart and Clark, 2003; Li and Tax, 2013; Dufayard et al., 2017). Later on the role of *LRR-RLKs* in pathogen sensing and activation of downstream defense response has been reviewed deeply (Nejat and Mantri, 2017). Due to the indispensable roles of *LRR-RLKs* in plants, they have been classified into two main classes (Diévart and Clark, 2003). First, the *LRR-RLK* is crucial for morphogenesis, organogenesis, hormone signaling, signifying development, and growth regulation. Secondly, numerous *LRR-RLK* members respond to biotic and abiotic stresses like Fusarium wilt, drought, salt, and cold, and hence are associated with defense (Afzal et al., 2008; Cao et al., 2020). Some of the *LRR-RLKs* have dual roles that might be because of the cross-talk among development and defense cascades or due to the binding of several ligands to a receptor (Afzal et al., 2008).

The *RPK1* gene is a calcium independent Serine-Threonine (Ser-Thr) kinase that belongs to the subfamily of *leucine-rich receptor kinases* (*LRR kinases*) and family of *Receptor-Like Kinases* (*RLK*) (Zou et al., 2014). *RPK1* is one of the short subfamilies with few genes that regulates abiotic stresses and root system architecture. The *RPK1* comprises of extracellular six LRR motifs, a transmembrane domain, extracellular ligand-binding domain, and single cytoplasmic kinase conserved domain in rice (Hong et al., 1997; Cheng et al., 2009; Motte et al., 2014). Studies in rice have shown that *RPK1* is involved in root system architecture (RSA) via regulating negatively polar auxin transport (PAT) and accumulation of auxin in roots (Zou et al., 2014). In other studies of rice, it was also reported that auxin defective mutants showed stunted growth and shorter roots (Uzair et al., 2021). Down-regulation of *RPK1* endorsed the growth and enhanced the height of the plant and number of tillers, whereas up-regulation resulted in immature lateral roots, adventitious roots, and a decreased apical meristem of roots (Zou et al., 2014). In *Arabidopsis*, the inhibition of *AtRPK1* displayed greater salt tolerance than normal plants, while overexpressed plants exhibited lesser salt tolerance degrees (Shi et al., 2014). The levels of *AtRPK1* were enhanced ominously under less water, abscisic acid (ABA), high salt and lower temperature (Hong et al., 1997). In *Arabidopsis thaliana*, inhibition of *RPK1* delayed ABA-induced senescence significantly (Lee et al., 2011). *AtRPK1* is also prerequisite for cotyledon primordial initiation of cotyledons during embryogenesis in *Arabidopsis thaliana* (Nodine et al., 2007; Nodine and Tax, 2008). *AtRPK1* positively regulates *CaM1* gene expression, which in turn regulates ROS (reactive oxygen species) production, leaf senescence, and ABA response (Dai et al., 2018).

*Triticum aestivum* L., commonly known as “bread wheat”, is a cereal and staple food grain all over the globe. Being a most consumed cereal crop, it was grown on a large scale of 240 million hectares in 2016 (Milner et al., 2018). However, due to water scarcity, nutrient deficiency, and abiotic stresses, wheat yield is curtailed (Mondal et al., 2015; Abbas et al., 2022). Wheat is a drought sensitive crop. Therefore, in order to meet the global demand, that is 50% of the grain in 20 years approximately, the varieties of wheat with effective utilization of minerals and water are requisite (Odegard and Van der Voet, 2014). Since roots are the main structures for the minerals and water uptake and decipher stress stimuli from soil (Fang et al., 2017). Hence, identification of stress-tolerant genes within the root system could be propitious.

Since the genome of *T. aestivum* has been sequenced, it is feasible to carry out a genome-wide analysis of different genes*.* In this study, 15 *TaRPK1* genes were analyzed for their structure, chromosomal location within the genome, phylogenetic relationships, conserved motifs, synteny, and *cis*-regulatory elements. Additionally, the patterns of expression of all 15 *TaRPK1* members were also studied *in silico*. RT-PCR expression analysis of *TaRPK1* members was also performed in Pakistan-13, Galaxy (drought tolerant), and Shafaq (drought susceptible) wheat varieties under normal and drought conditions. The current study enlightens the role of *TaRPK1* genes in plant developmental processes under drought conditions and provides a solid foundation for the functional characterization of the wheat *RPK1* gene family.

## Materials and Methods

### Identification of *RPK* Gene Family Members in *T. aestivum*

The sequence IDs of *Arabidopsis* and rice *RPK1* genes were acquired from the available literature (Shi et al., 2014; Zou et al., 2014). These sequences were retrieved from Ensembl plants and NCBI, which were then used as queries for the Basic-Local Alignment Search tool (BlastP and BlastN) against IWGSC (INSDC Assembly GCA_900519105.1 July 2018 database version 106.4), NCBI (https://www.ncbi.nlm.nih.gov/) and Ensembl plants (plantshttps://plants.ensembl.org/index.html) for *T. aestivum*. For all of the candidate *RPK1* genes, the kinase domain presence was substantiated with Pfam (http://pfam.sanger.ac.uk), and by SMART (http://smart.embl-heidelberg.de/) (Letunic and Bork, 2018) databases. The sequences in which the kinase domain was absent were removed (Supplementary Table S1). *In silico* based putative protein information of *RPK1* genes (physio-chemical) was analyzed through the Protparam (https://web.expasy.org/protparam/) tool. The subcellular localization of RPK1 proteins was predicted via Plant-mSubP and pLoc-mPlant (http://bioinfo.usu.edu/Plant-mSubP/ ; http://www.jci-bioinfo.cn/pLoc-mPlant/ ) (Cheng et al., 2017; Sahu et al., 2020).

### Chromosomal Location of *TaRPK1* Genes

The chromosomal locations of all candidate *RPK1* genes in *T. aestivum* were acquired from Ensembl (http://plants.ensembl.org/Triticum_aestivum/Info/Index). The gene map of *TaRPK1* genes was drawn with the help of MapChart and confirmed through TBtools.

### Phylogenetic Analysis of RPK1 Proteins

To retrieve the RPK1 protein sequences, the amino acid sequences of 15 TaRPK1 members were used as queries to blast (BLASTP) against the *Triticum turgidium*, *Triticum dicoccoides*, *Titicum urartu*, *Triticum speltoides*, *Aegilops tauschii*, *Hordeum vulgare*, *Arabidopsis thaliana,* and different species of *Oryza* (*rufipigon*, *japonica*, *indica,* and *glaberrima*). The sequences with > 60% identities were retrieved from Ensembl (http://plants.ensembl.org). The phylogenetic trees were made by means of MEGA-X software with NJ (neighbor-joining method) (Kumar et al., 1994). The parameter Poisson model and pairwise deletion were used with replicates of 1,000 bootstraps for assessment of node significance.

### Prediction of Gene Structure and Conserved Motifs in TaRPK1 Proteins

The number of exons and introns was predicted by the gene structure display server (GSDS, http://gsds.cbi.pku.edu.cn/) and the genomic sequences and coding sequences were aligned using ClustalW. Conserved motifs in RPK1 proteins of *T. aestivum* were analyzed using MEME, a multiple-EM for motif elicitation program (http://meme-suite.org/tools/meme) (Bailey et al., 2009). The execution of MEME search was done with default parameters apart from motif maximum number, which was set to 10, and optimum motif width of ≥6 and ≤200 was selected.

### Gene Ontology Enrichment Analysis

The analysis of *TaRPK1* gene ontology was performed by TaRPK1 protein sequences via the online gProfiler tool (https://biit.cs.ut.ee/gprofiler/gost) with default parameters (Raudvere et al., 2019).

### miRNA Prediction in Wheat *RPK1* Family Genes

The miRNA prediction was performed as mentioned formerly (Yan et al., 2019). The *TaRPK1* sequences were submitted for potential miRNA prediction through a search against the available wheat miRNA reference by means of the psRNATarget Server (https://www.zhaolab.org/psRNATarget/), using default settings (Dai and Zhao, 2011). The visualization of the interaction network of the predicted miRNA with their corresponding *TaRPK1* target genes was done by Cytoscape software (https://cytoscape.org/) with default settings (Shannon et al., 2003).

### Interpretation of Putative Regulatory *Cis*-Acting Elements

The sequence size of 2 kb in the upstream region were dug out from all *TaRPK1* genes of *T. aestivum* that acted as promoters for the regulatory *cis* acting elements prediction through the PlantCare (http://bioinformatics.psb.ugent.be/webtools/plantcare/html/) database (Lescot et al., 2002).

### Collinearity Prediction and Synteny Analysis

The GFF3 files and proteomes of *Triticum aestivum* and its ancestors, including *Aegilopes tauschii, Triticum spelta*, *Triticum turgidum,* and *Triticum dicoccoides,* were used from the Ensembl Plants database for collinearity prediction via the MCScanX algorithm (Wang et al., 2012). Synteny scrutiny of *RPK1* family members was performed via Tbtools (Chen et al., 2018).

### Three-Dimensional Protein Structure Prediction

The TaRPK1 protein structures were modeled via amino acid sequence using the SWISS-MODEL database (https://www.swissmodel.expasy.org) (Biasini et al., 2014), and for visualization of 3D structure Pymol software (https://pymol.org/2/) was applied. The verification and validation of the predicted 3D structures of TaRPK1 proteins were assessed using the Ramachandran Plot—Zlab, (https://zlab.umassmed.edu/bu/rama/) (Anderson et al., 2005).

### *In Silico* Differential Expression Patterns of *RPK* Genes

*In silico* expression analysis was performed using the wheat-expression browser (www.wheat-expression.com) at different wheat stages (Kaur et al., 2017). The data were unruffled in the course of developing seedling, vegetative, and reproductive stages from different organs of wheat such as roots, leaf sheath, leaf blade, shoot, spike, and grain. The heatmap was then created from the composed data, based on the expression values of genes (in TPM) by means of Tbtool.

### Interaction Network and Co-Expression Analysis

For interaction network studies, String (https://string-db.org/) was used by selecting *Triticum aestivum* as a platform species. For visualization of the molecular library, Cytoscape was used. Correlation coefficients on the basis of verities, treatments, and tissues were calculated in R 3.4.0. These coefficients indicate the degree of association among the terms and provide linkages among the *TaRPK1* members.

### Plant Material and Stress Treatment

Previously, Pakistan-13, Galaxy, and Shafaq were studied under drought stress and categorized as drought tolerant and susceptible varieties, respectively (Shabbir et al., 2015; Ulfat et al., 2017; Ahmad et al., 2021; Wasaya et al., 2021; Iqra et al., 2022). So, seeds of these varieties were obtained and sown under controlled glass-house conditions at the National Institute for Genomics and Advanced Biotechnology (NIGAB), National Agriculture Research Center (NARC), Islamabad, Pakistan. After 2 weeks of sowing (seedling stage), the roots and leaves tissues were collected. At growth stage 8 (tillering stage), roots, stems, and leaf tissues were collected. At the grain filling stage (14 days after flowering), sampling for roots, stems, leaf, and grains was done (Hyles et al., 2020). For expression profile analysis under drought stress, seeds of selected varieties were first surface sterilized with sodium hypochlorite followed by three washings, then soaked in distilled water in a growth chamber (16 h light/8 h dark cycle at 22°C). After 2 weeks, young seedlings were treated with 20% polyethylene glycol (PEG) 6,000 (v/v). The root and leaf tissues of seedlings were harvested after 12 h of exposure to stress conditions. All the samples were collected in three replicates, and samples were frozen immediately in liquid nitrogen, and placed in −80°C storage for RNA extraction.

### RNA Isolation and qRT-PCR Analysis

Approximately 100 mg of tissues were taken for total RNA extraction using an RNA mini kit (Cat # 12183018A, Invitrogen, Thermo Fischer Scientific) followed by the manufacturer’s instructions. Through agarose gel electrophoresis, the quality and concentration of RNA were determined, followed by optical density measurement through a spectrophotometer. With the help of the RT Prime-Script Reagent Kit, the cDNA was made from 1 ug of RNA. Specific primers were designed for *TaRPK1* genes manually, followed by confirmation via NCBI Primer Blast software (http://www.ncbi.nlm.gov/tools/primer-blast), provided in Supplemental Table S2. The qRT-PCR was accomplished with SYBR Green I (Roche) Master Mix. Wheat *β-Actin* was used as a control reference gene. Three independent biological replicates were analyzed for each sample. The values were means and standard deviations (SD) were calculated from biological replicates. The relative expression levels of each gene were studied by means of 2^−∆∆Ct^ (Schmittgen and Livak, 2008).

## Results

### Analysis and Sequence Identification of *RPK1* Genes in *T*. *aestivum*

A set of 15 candidate *RPK1* genes were retrieved from *Triticum aestivum* based on BlastP and BlastN. A domain search by the SMART tool with the corresponding RPK1 candidate amino acid sequences confirmed the S_TKc Domain (SM00220). Thus, a total of 15 *TaRPK1* with complete structures were analyzed in *T. aestivum* (Table 1). Subsequent sequence identification of 15 TaRPK1 showed the protein length of 609–1,123 amino acids and a molecular mass ranged from 67–120 kDa. The iso-electric points (PI) of these proteins were 6–9. The Instability Index (II) ranged from 28.37–52.17, the Aliphatic Index (AI) was 87.93–106.38, and the grand-average of hydropathicity (GRAVY) −0.032–0.138. The instability index of group I was less than 40, representing stable proteins, whereas proteins of groups II and III showed instability index values of more than 40, indicating unstable proteins. The AI signified that all of the TaRPK1 proteins are thermally stable. The GRAVY indicated TaRPK1 proteins to be hydrophilic proteins except for TaRPK10, TaRPK11, and TaRPK12, which showed a value less than zero, representing them as hydrophobic proteins. The sub-cellular localizations of the TaRPK1 were anticipated, which showed that all the TaRPK1 were localized to the cell membrane (Table 2).

**TABLE 1:** *In silico* prediction of identified *RPK1* genes in wheat and sequence characteristics.

Sr. no.	New name	Gene ID	Chr No.	Chr	Orientation	CDS (bp)	No. of exons	Coding exons	No. of introns
				location					
1	TaRPK1.1 (TaRPK1)	TraesCS1A02G304200	1A	497,503,763–497,509,507	R	2,895	19	19	18
2	TaRPK1.2 (TaRPK2)	TraesCS1B02G314700	1B	539,546,762–539,552,423	R	2,895	19	19	18
3	TaRPK1.3 (TaRPK3)	TraesCS1D02G303700	1D	401,666,525–401,672,077	R	2,895	19	19	18
4	TaRPK1.4 (TaRPK4)	TraesCS3A02G340100	3A	587,403,291–587,408,585	F	2,676	17	17	16
5	TaRPK1.5 (TaRPK5)	TraesCS3B02G371700	3B	584,546,469–584,551,744	F	2,676	17	17	16
6	TaRPK1.6 (TaRPK6)	TraesCS3D02G333600	3D	445,633,883–445,639,177	F	2,676	17	17	16
7	TaRPK1.10 (TaRPK10)	TraesCS2A02G176500	2A	136,053,228–136,056,887	R	3,372	2	1	0
8	TaRPK1.11 (TaRPK11)	TraesCS2B02G202900	2B	182,708,242–182,711,907	R	3,372	2	1	0
9	TaRPK1.12 (TaRPK12)	TraesCS2D02G183900	2D	129,186,794–129,190,494	R	2,949	2	2	1
10	TaRPK1.13 (TaRPK13)	TraesCS2A02G260600	2A	410,851,518–410,855,096	F	2,187	2	1	0
11	TaRPK1.14 (TaRPK14)	TraesCS2B02G281400	2B	388,595,342–388,597,540	R	1830	2	2	1
12	TaRPK1.15 (TaRPK15)	TraesCS2D02G263100	2D	320,280,150–320,283,723	R	2,199	2	1	0
13	TaRPK1.7 (TaRPK7)	TraesCS3A02G340000	3A	587,396,690–587,401,627	F	2,772	18	18	17
14	TaRPK1.8 (TaRPK8)	TraesCS3B02G371600	3B	584,539,043–584,544,883	F	2,874	19	19	18
15	TaRPK1.9 (TaRPK9)	TraesCS3D02G333500	3D	445,627,122–445,632,371	F	2,775	18	18	17

Chr, chromosome; F, forward strand; R, reverse strand; CDS, coding sequence; bp, base pairs.

**TABLE 2:** *In silico*-based putative protein information of *RPK1* genes identified in *T. aestivum.*

Sr. no.	New name	Sequence ID	PL (Aa)	Domain loc	Mol. wt. (Kda)	pI	II	AI	GRAVY	SCL
1	TaRPK1.1 (TaRPK1)	TraesCS1A02G304200	964	632–901	104	8	28.37	91.76	-0.032	Cell membrane
2	TaRPK1.2 (TaRPK2)	TraesCS1B02G314700	964	632–901	104	8	29.41	91.05	-0.046	Cell membrane
3	TaRPK1.3 (TaRPK3)	TraesCS1D02G303700	964	632–901	104	8	30.18	90.54	-0.04	Cell membrane
4	TaRPK1.4 (TaRPK4)	TraesCS3A02G340100	891	563–832	97	6	37.72	89.55	-0.108	Cell membrane
5	TaRPK1.5 (TaRPK5)	TraesCS3B02G371700	891	563–832	97	6	38.74	88.99	-0.109	Cell membrane
6	TaRPK1.6 (TaRPK6)	TraesCS3D02G333600	891	563–832	97	6	36.85	89.33	-0.099	Cell membrane
7	TaRPK1.10 (TaRPK10)	TraesCS2A02G176500	1,123	841–1,048	120	8	42.51	103.46	0.121	Cell membrane
8	TaRPK1.11 (TaRPK11)	TraesCS2B02G202900	1,123	841–1,112	120	7	42.79	104.32	0.138	Cell membrane
9	TaRPK1.12 (TaRPK12)	TraesCS2D02G183900	982	700–971	104	8	42.82	106.38	0.135	Cell membrane
10	TaRPK1.13 (TaRPK13)	TraesCS2A02G260600	728	499–720	80	9	52.17	101.35	-0.063	Cell membrane
11	TaRPK1.14 (TaRPK14)	TraesCS2B02G281400	609	330–601	67	9	47	98.74	-0.054	Cell membrane
12	TaRPK1.15 (TaRPK15)	TraesCS2D02G263100	732	453–724	80	9	51.16	100.26	-0.035	Cell membrane
13	TaRPK1.7 (TaRPK7)	TraesCS3A02G340000	923	593–863	101	7	31.67	89.51	-0.147	Cell membrane
14	TaRPK1.8 (TaRPK8)	TraesCS3B02G371600	957	627–897	104	7	31.92	90.2	-0.146	Cell membrane
15	TaRPK1.9 (TaRPK9)	TraesCS3D02G333500	924	594–864	101	7	31.27	87.93	-0.179	Cell membrane

Loc, location; Mol. wt., molecular weight; pI, isoelectric point; II, instability index; AI, aliphatic index; GRAVY, grand average of hydropathicity; SCL, sub-cellular localization.

A detailed protein alignment of structural predictions showed that all TaRPK1 proteins are composed of the leucine-rich repeat N terminal (LRRNT_2) domain, leucine-rich repeat (LRR) domains, transmembrane domain I, and a serine–threonine kinase (S_TKc) domain. However, the LRR domains were missing in TaRPK1, TaRPK2, and TaRPK3 sequences (Supplemental Figure S1).

### Chromosomal Distribution of *RPK* Genes

**FIGURE 1:**
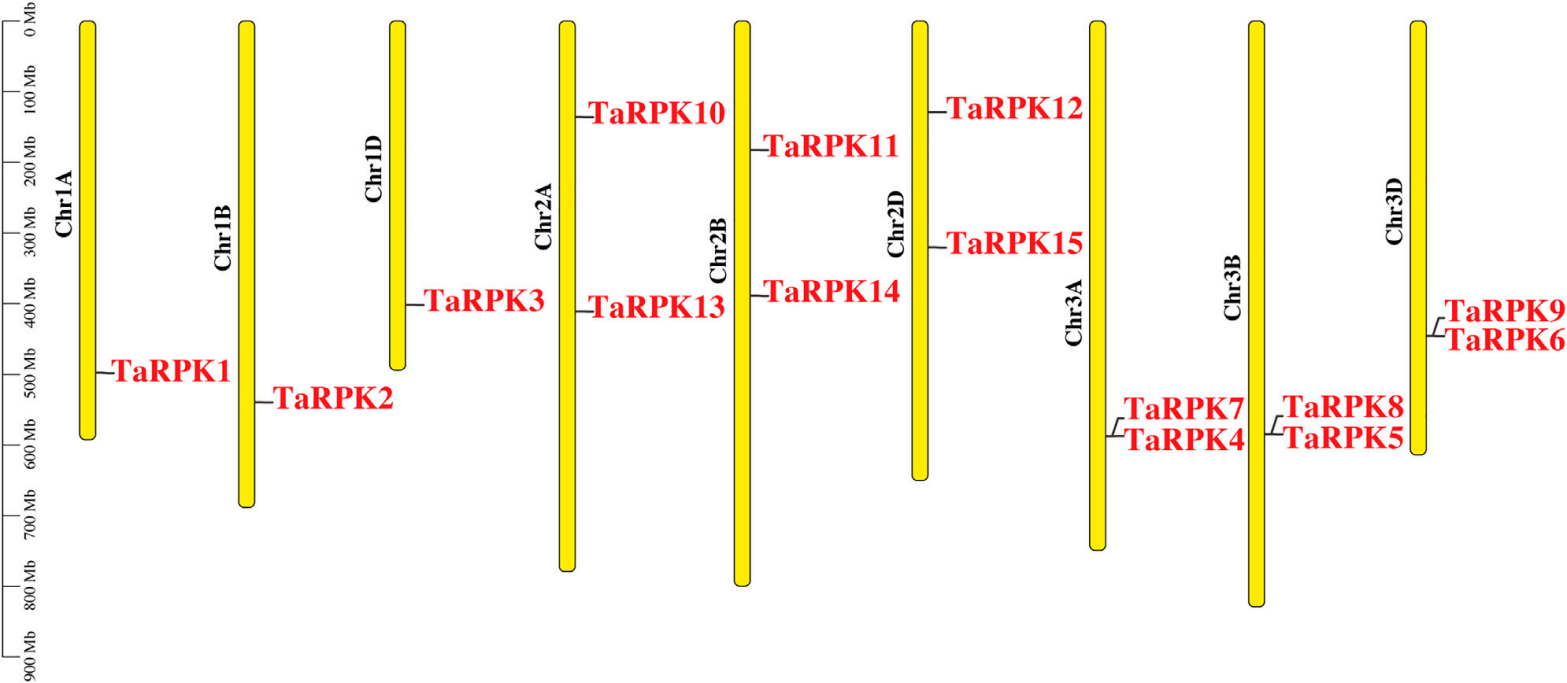
Chromosomal location of *T. aestivum RPK* genes on chromosomes in A, B*,* and D genomes. Respective chromosome numbers are written as Chr IA to Chr 3D on the top of each chromosome. Gene position can be estimated using the scale (in megabase; Mb) on the left of the figure.

The physical location of *RPK* genes in *T. aestivum*, to the corresponding chromosomes, is shown in Figure 1. A total of 15 *RPK* genes were mapped on 9 out of 21 chromosomes in wheat. The genes were mainly mapped on chromosomes 1, 2, and 3 on the respective A, B, and D genomes. No *RPK* genes were found on the rest of the chromosomes.

### Phylogenetic Analysis of TaRPK1 Proteins

**FIGURE 2:**
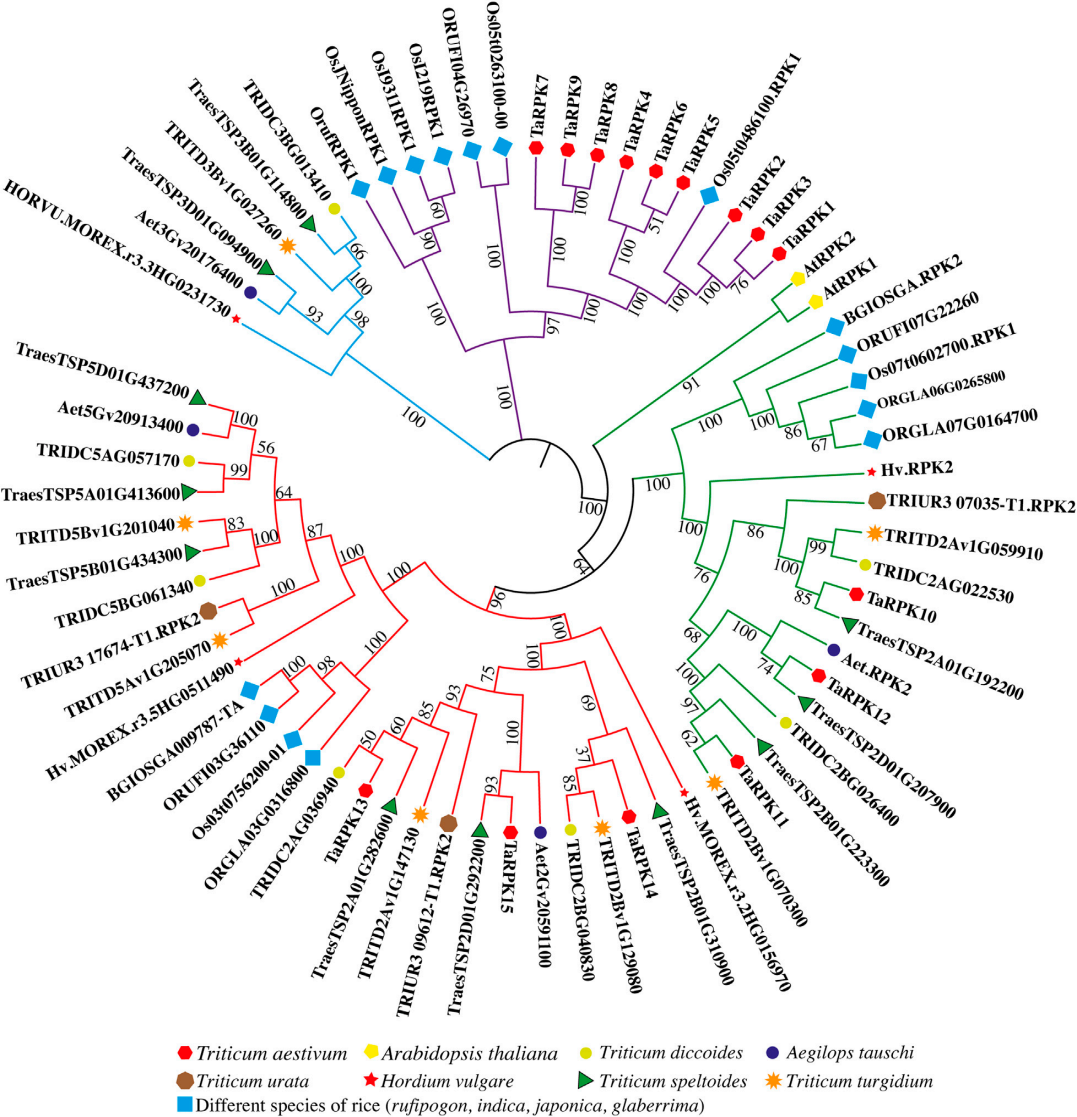
Comparative phylogenetic tree of *RPK* genes between *Triticum aestivum*, *Triticum dicoccoides*, *Triticum turgidum*, *Triticum speltoides*, *Aegilops tauschii*, *Triticum urata*, *Hordeum vulgare*, *Arabidopsis thaliana,* and different species of *Oryza* (*rufipigon*, *indica*, *japonica*, and *glaberrima*). 1,000 replicates were used for the bootstrap test, and the replication percentage is presented next to the branches.

Of the 15 identified *TaRPK1* genes in this study in *Triticum aestivum*, two *RPK* genes from *Arabidopsis thaliana*, 16 *RPK* genes from rice, seven *RPK* genes from *Triticum dicoccoides*, three *RPK* genes from *Triticum urata*, seven *RPK* genes from *Triticum turgidium*, four *RPK* genes from *Aegilops tauschii*, 11 *RPK* genes from *Triticum speltoides,* and four *RPK* genes from *Hordeum vulgare* were used to construct a neighbor-joining based tree with MEGA X software in order to study the evolutionary relationships (Figure 2). The phylogenetic tree generated on the basis of similarities with protein sequences distributed *RPK* members into four main groups, with *TaRPK1* members in three groups. Overall group I possessed nine *TaRPK1* members (*TaRPK1-9*), that were closely associated with *RPK* members of rice. Group II (*TaRPK10-12*) and Group III (*TaRPK13-15*) exhibited three *TaRPK1* members each, that exhibited close association with *Triticum turgidium, Triticum speltoides, Aegilops tauschii* and *Triticum dicoccoides.*

### Analysis of *TaRPK1* Gene Structure and Conserved Motif

**FIGURE 3:**
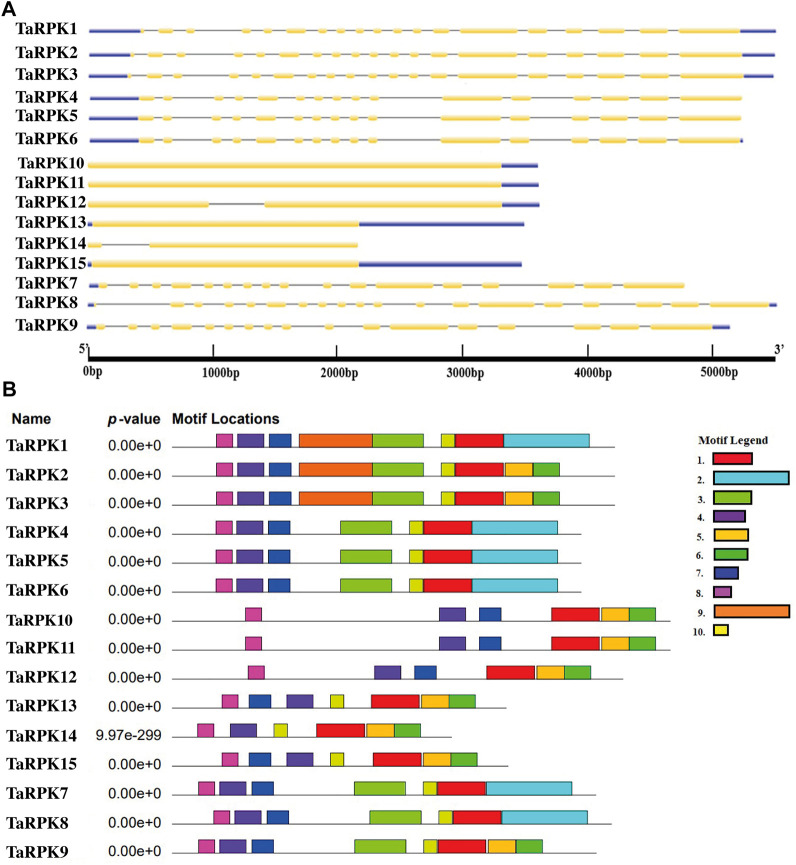
**(A)** Exon–intron structures of *T. aestivum RPK1* genes. Yellow boxes denote exons, straight black lines represent introns, and blue boxes denote upstream/downstream. **(B)** Schematic representation of identified motifs in *T. aestivum* RPK1 proteins using the MEME motif search tool. Different colors indicate different motifs.

The intron–exon number and arrangements of the *RPK1* members were envisaged through comparing the coding sequence with the genomic DNA sequence. All of the *TaRPK1* genes in group I consisted of 16–18 introns, except for the groups II and III that contained 0 and 1 intron (Figure 3A). Furthermore, the conserved motifs within TaRPK1 proteins were predicted by online MEME software. Ten conserved motifs (1-10) were analyzed (Figure 3B and Supplementary Figure S2). The motifs 1, 3, 4, 7, 8, and 10 were present in all of the *RPK1* sequences. However, group II did not display motifs 3 and 10, and the motif three was also missing in group III sequences.

### Gene Ontology of *RPK1* Genes

**FIGURE 4:**
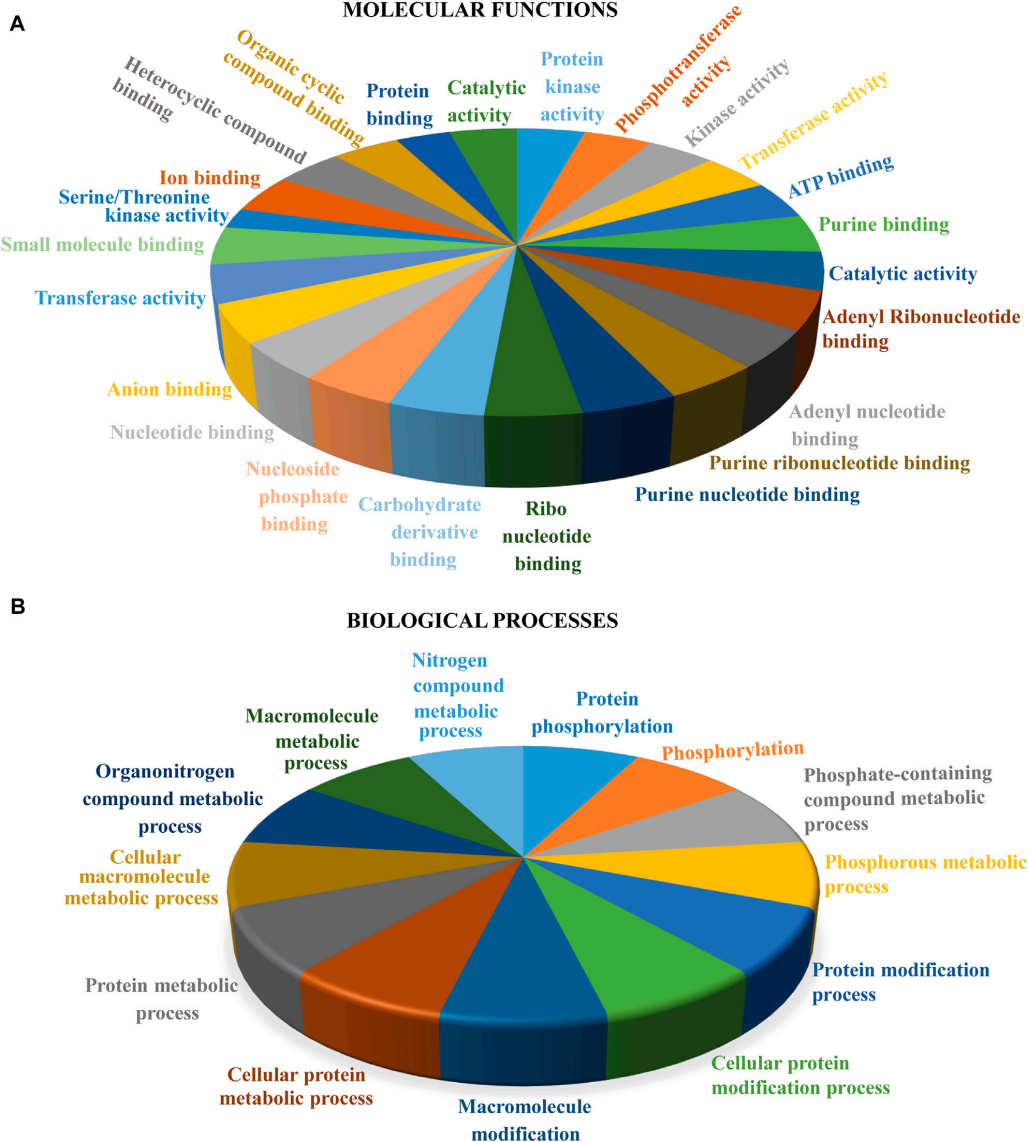
Gene Ontology prediction of *RPK1* genes. The data represent **(A)** molecular functions and **(B)** biological processes.

GO annotation analysis was conducted for the functional analysis of *RPK1* genes. *In-silico* functional prediction was performed, and the results displayed two types of processes involved, that is, molecular processes (MPs) and biological processes (BPs) (Figure 4 and Supplementary Table S3). Biological processes indicate that *RPK* members are involved actively in various metabolic processes. The molecular processes suggested the *RPK1* member’s catalytic activity. Such outcomes clearly denote *RPK1* genes’ significant role in growth and development via modulation of molecular and biological processes.

### MicroRNA Targeting *TaRPK1* Genes

**FIGURE 5:**
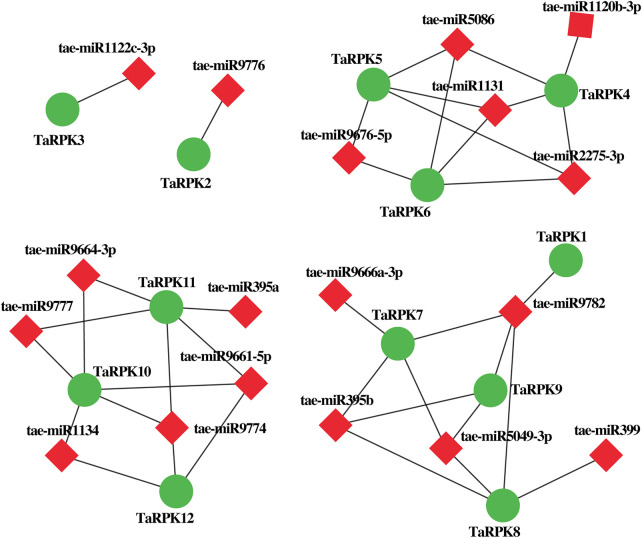
MicroRNA and their targeted *TaRPK1* genes. Regulatory network relationship between miRNA and their targeted *TaRPK1* genes.

We also identified putative 18 miRNAs targeting *TaRPK1* genes for the generation of interaction networks by Cytoscape software in order to better understand the underlying miRNA mechanism involved in the modulation of *TaRPK1* genes (Figure 5 and Supplementary Table S4). In the connection distribution and regulation network, *TaRPK1*, *TaRPK2,* and *TaRPK3* were found targeted by single miRNAs, which are tae-miR9782, tae-miR9776, and tae-miR1122c-3p, respectively. *TaRPK10* and *TaRPK11* are the most targeted *RPK1* wheat genes by tae-miR1134, tae-miR9774, tae-miR9661-5p, tae-miR9664-3p and tae-miR9777 targeting *TaRPK10,* and tae-miR9774, tae-miR9777, tae-miR9664-3p, tae-miR395a and tae-miR9661-5p targeting *TaRPK11* genes. However, no miRNA was found targeting *TaRPK13*, *TaRPK14*, and *TaRPK15* genes.

### Regulatory *Cis*-Element Interpretation in *T. aestivum*

**FIGURE 6:**
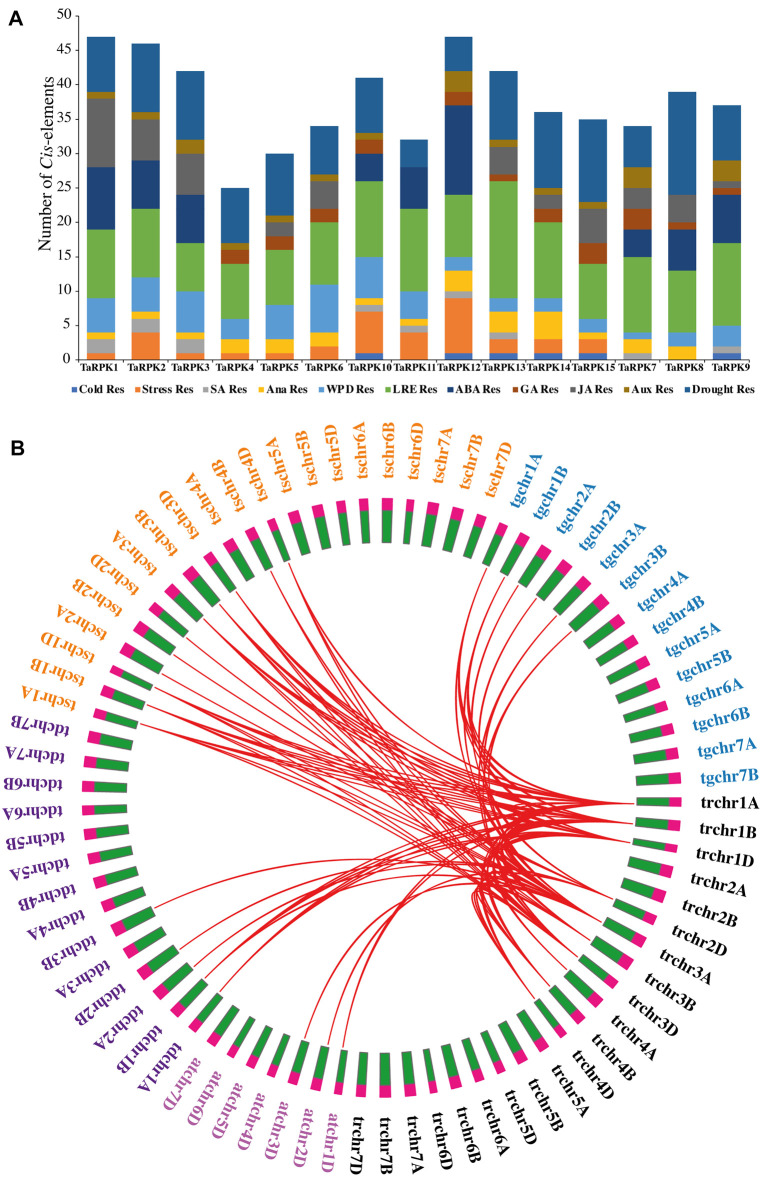
*Cis*-Elements and evolutionary conservation. **(A)** Regulatory *Cis*-element prediction of 2-Kb sequence upstream of *RPK1* genes in *T. aestivum.* **(B)** Syntenic relationship between *Triticum aestivum* (*tr,* black), *Triticum dicoccoides* (*td*, purple), *Triticum turgidum* (*tg*, blue), *Aegilops tauschii* (*at,* pink), and *Triticum speltoides* (*ts*, orange).

The promoter regions contain *cis*-modulatory elements which are critical for the binding of transcription factors for transcription initiation, which has an essential function in the expression of genes. The promoter regions of *RPK1* members were used for the *cis*-regulatory element prediction (Figure 6A). The results indicated that the *cis*-regulatory elements can be distributed into several categories, such as hormone related elements, light-related elements, developmental responsive elements, abiotic stress responsive elements, promoter-related motifs, and other motifs. Amid them, the elements chiefly present were associated with photoreaction, hormone responsiveness, and abiotic stress-related motifs. The photoreaction responsive cis-regulatory elements included ACE, AE-Box, ATCT, G-Box, GATA, GT1, SP1, AT1, Box 4, Box II, I-Box, TCT, GA, L-Box, TCCC, and ATC motif. The most abundant light-responsive elements were found in *TaRPK11* and *TaRPK13,* which had 17 and 12 members, respectively. Hormone responsive elements were also copiously present in the *RPK1* promoter, mostly comprising abscisic acid response elements. The three extensively distributed cis elements were related to abiotic stress response, among which drought responsive elements were profuse. Other elements correlated to abiotic stress were also identified.

### Syntenic Relationship Analysis

In order to understand the evolutionary relationship and origin of *Triticum aestivum* (tr) with *Triticum turgidum* (tg), *Aegilops tauschii* (at), *Triticum speltoides* (ts) and *Tritium dicoccoides* (td), a comparative synteny scrutiny of RPK protein sequences was performed. The proteins were closely related among five species and exhibited significant similarity in analysis of evolutionary correlation. It was observed that the *TaRPK1* genes of *T. aestivum* have similar origins of evolution to other *Triticum* species (Figure 6B and Supplementary Table S5).

### *In silico* 3D-Structure Prediction of TaRPK1 Proteins

**FIGURE 7:**
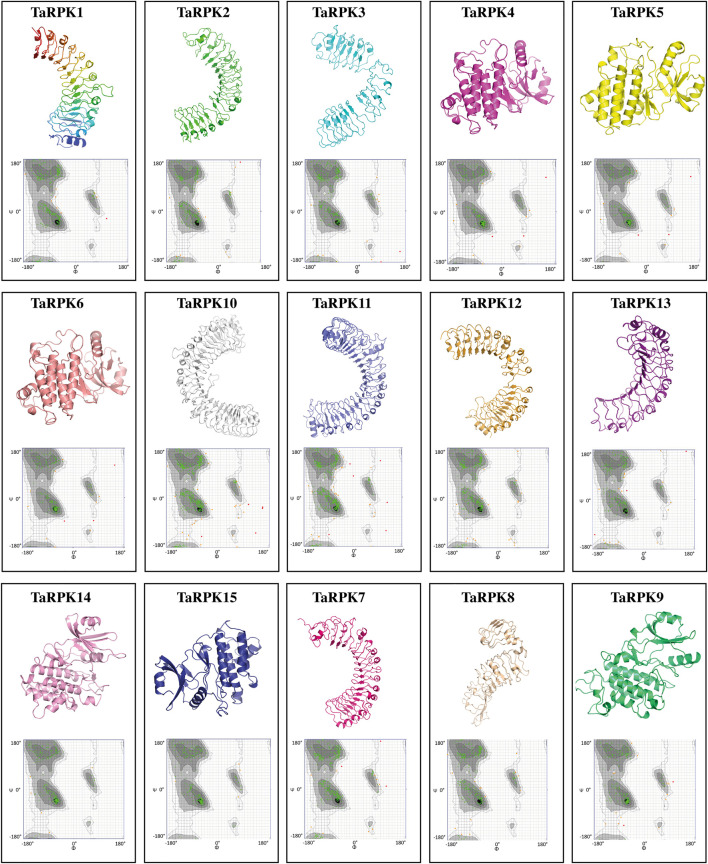
3D structure of *TaRPK1* proteins along with Ramachandran plots in *T. aestivum*. In all 3D protein structures, the spirals are helices, broad strips with arrow heads are beta-pleated sheets, and thin loops are coils. In Ramachandran plots, dark black, gray, and light gray represent highly preferred conformations with Delta ≥ −2. White with a black grid denotes preferred conformations with −2 > Delta ≥ −4. White with gray grid symbolizes questionable conformations with Delta < −4. The green crosses signify highly preferred observations, brown triangles specify preferred observations, and red circles represent unfavorable observations.

Three-dimensional (3D) structures of TaRPK1 proteins were predicted by using SWISS_MODEL online computational software. 3D structures of target proteins were anticipated based on homology modeling. The SWISS MODEL predicted 15 successful models of TaRPK1 proteins with at least 30% identity to the template (4mn8.1. A, 5hyx.1. A, 5xkj.1. C, 6mOu.1. A, 4mna.1. A, 4oh4.1. A, 6cth.1. A, 7brc.1. A, and 5tos.1. A) that was a widely recognized threshold for effective modeling (Xiang, 2006). However, TaRPK2 and TaRPK3 showed sequence identity of 27.84% and 29.47%, respectively, with the template, which was less than 30%. The highest sequence identity of 45% with the template was observed by TaRPK4, TaRPK5 and TaRPK6 (Figure 7). The verification and validation of the predicted 3D structure of TaRPK1 were assessed via Ramachandran Plots (Anderson et al., 2005) that validated the backbone diahedral angles of the targeted protein. The Ramachandran plot assessment showed that 92–98% of the regions of TaRPK1 protein showed highly favorable regions, which indicates the stability and good quality of the predicted protein structure (Supplemental Table S6).

### Genome Wide Expression Patterns of *RPK* Genes

**FIGURE 8:**
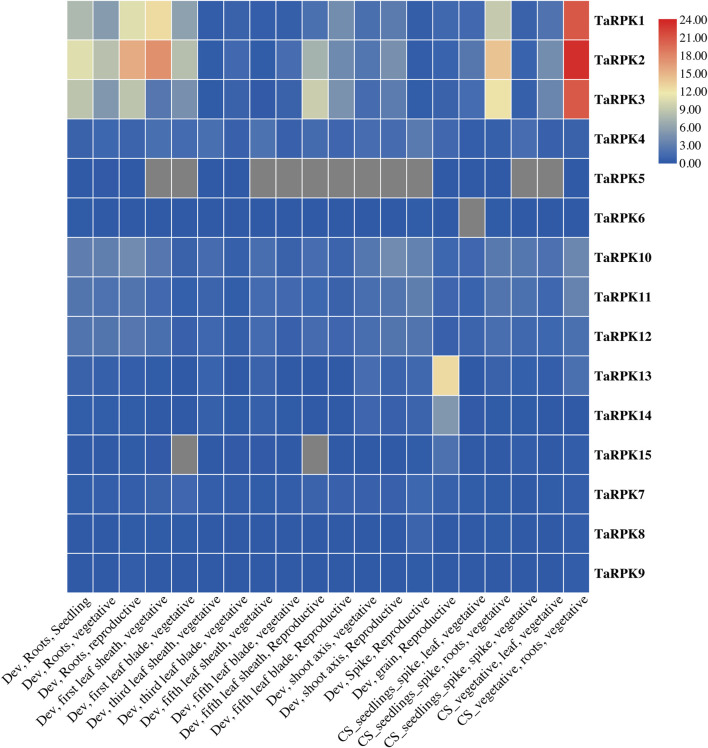
*In silico* analysis of *RPK* members in different tissues (root, shoot, leaf, grain, and spike) was generated using TB tool. Dev = developmental stage, CS = seedlings spike; Chinese Spring seedlings (leaves, roots) and spikes at anthesis and CS vegetative; Chinese Spring leaves and roots from seven leaf stages. The heatmap was constructed from transcripts per 10 million values with the scale bar displaying expression of the genes. The blue and red colors denote lower and higher expressions of the transcripts, respectively.

The data of RNA-seq for all of the 15 *RPK* sequences were obtained from online database. A heatmap was generated showing expression levels of *RPK* members at different stages, namely seedling stage, vegetative stage, and reproductive stage (Figure 8 and Supplementary Table S7) and in various organs (root, leaf, shoot, spike, and grain) of wheat. The highest expression of *TaRPK1* members was observed in root tissues compared to other tissues. *TaRPK1, TaRPK2,* and *TaRPK3* exhibited the highest expression patterns in roots at seedling, vegetative, and reproductive stages. Higher to moderate expression was observed in grain at the developing reproductive stage by *TaRPK13* and *TaRPK14*, respectively. Spikes, leaves, and shoots showed moderate to low expression in all of the *TaRPK1* members in wheat.

### Expression Analysis of *RPK1* Genes in *T*. *aestivum*

**FIGURE 9:**
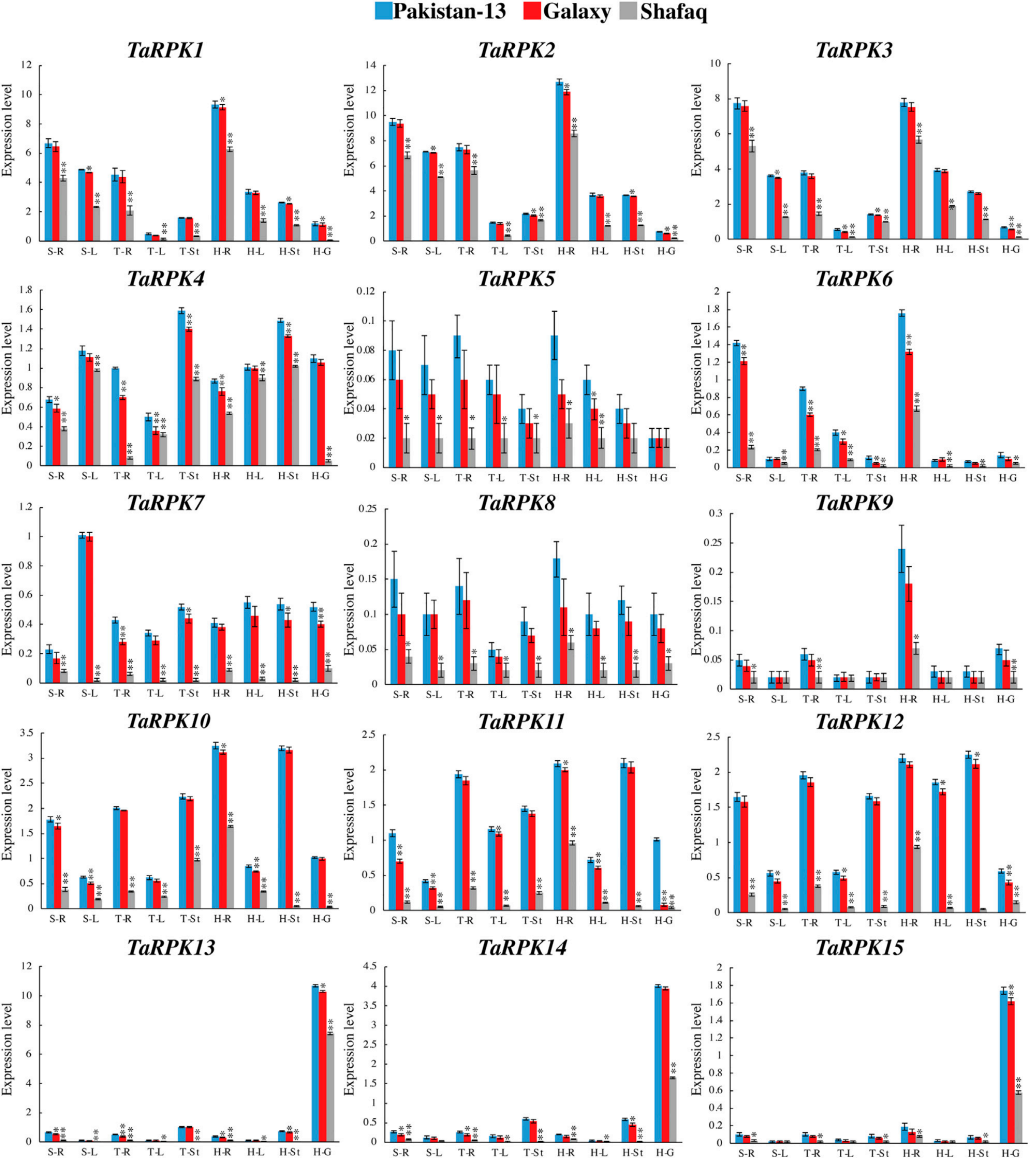
Real time PCR-expression analysis of *TaRPK1* genes in wheat varieties; Pakistan 13 (blue), Galaxy (red), and Shafaq (gray). The wheat plant was germinated and grown in soil under normal conditions. Expressions of *TaRPK1* genes were determined in root (R), stem (St), leave (L), and grain (G) at seedling (S), tillering (T), and heading (H) stages. Standard deviation (SD) of three biological replicates is represented by the error bars. Significance was assessed by using a *t*-test (*p < 0.05, **p < 0.01, and ns = non-significant).

The *TaRPK1* gene expression was determined in drought-tolerant (Pakistan 13 and Galaxy) and drought-susceptible (Shafaq) varieties under normal growth conditions in order to get a baseline expression profile. The expression pattern in all of the three varieties was examined in various developmental stages, including seedling stage, tillering stage, and heading stage and in different tissues such as root, stem, leaf, and grain (Figure 9). The *TaRPK1*, *TaRPK2,* and *TaRPK3* showed significant expression in the roots at the heading and seedling stages of the Pakistan-13 and Galaxy varieties. The *TaRPK13* exhibited higher expression in grain tissues of all varieties compared to other *TaRPK1*. The *TaRPK1* genes displayed higher expression in roots whereas they showed less expression in leaves and stems compared to the grain and root expression in developmental stages. Our results indicated that *TaRPK1* genes had similar expression patterns in both Pakistan 13 and Galaxy varieties, unlike the Shafaq variety. The higher expression of *TaRPK1* genes was observed in the heading > seedling > tillering stages in Pakistan 13, Galaxy, and Shafaq varieties. Overall, *TaRPK1* exhibited significant expression in root tissues compared to leaf, shoot, and grain tissues.

**FIGURE 10:**
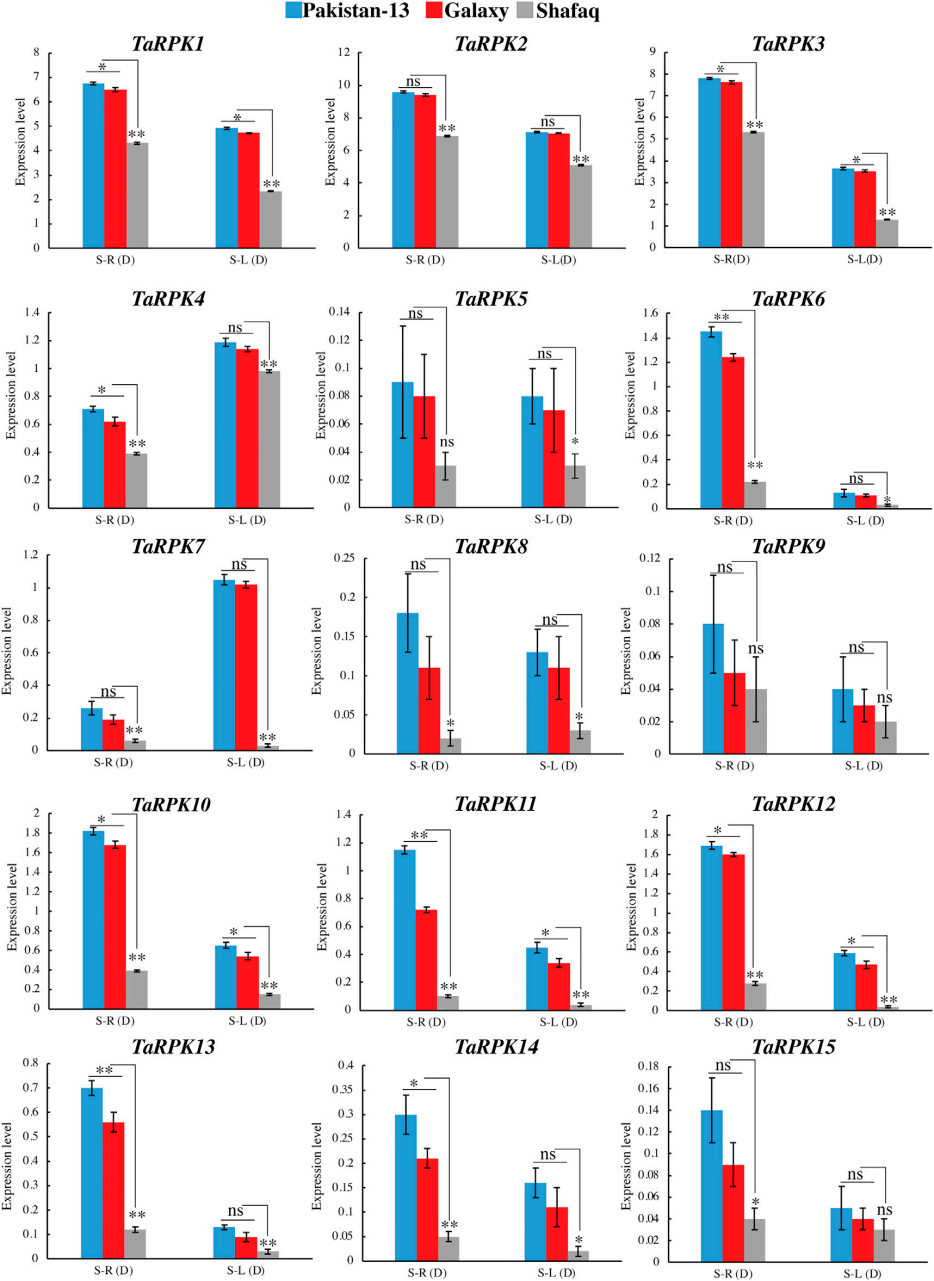
Expression profiling of *TaRPK1* genes under 20% PEG stress in Pakistan 13, Galaxy, and Shafaq varieties. Expressions of *TaRPK1* genes were determined in root (S–R) and leave (S–L) at the seedling stage (S). Error bars denote standard errors of three biological replicates. Significance was assessed by using a *t*-test (*p < 0.05, **p < 0.01, and ns = non-significant).

Roots are a good source to study the drought mechanism. To further confirm this, qRT-PCR showed the expression of *TaRPK1* members in the leaves and roots of two-week-old seedlings with drought stress through PEG simulation. PEG-6000 treatment induced an upregulated expression in roots and leaf tissues in comparison to the susceptible genotype. Higher expression was observed in root seedlings in comparison to the leaf seedlings, except for *TaRPK4* and *TaRPK7*, where higher expression was detected in the leaf tissues compared to the root tissues under drought stress (Figure 10). The *TaRPK1* genes displayed higher expression in Pakistan 13 > Galaxy > Shafaq varieties. Furthermore, we also performed co-expression (Supplementary Figure S3) and interaction network (Supplementary Figure S4 and Supplementary Table S8) analyses and the results revealed that all the *RPK1* members showed highly significant associations. These results indicate *TaRPK1* gene involvement in drought stress regulation.

## Discussion

*RPK1* is a serine/threonine protein kinase and belongs to the subfamily LRRKs, which is the largest subfamily of RLK. The LRRKs play a crucial role in a large number of biological activities, from development and growth to stress management in plants (Dufayard et al., 2017). *RPK* genes play significant roles in root system architecture (RSA), plant height, number of tillers, salt tolerance, cotyledon primordial initiation of cotyledons during embryogenesis, ABA-induced senescence, and ROS production (Shi et al., 2014; Zou et al., 2014; Dai et al., 2018). The functional characterization of *RPK1* members in wheat has not been reported in previous studies. The reason for it could be a complex allohexaploid (2n = 6x = 42) genome and other factors. Since *RPK1* genes are accountable for essential roles in plants, therefore a comprehensive study was performed to determine the chromosomal location, phylogenetic analysis, gene structure and expression of these genes in *T. aestivum*.

The standard process for the sequence identification of a new gene family is by a BLAST search of sequences of known proteins in model plants. A similar BLAST method was used to characterize two *RPK* genes from *Arabidopsis thaliana*, 16 *RPK* genes from different species of rice, seven *RPK* genes from *Triticum dicoccoides*, three *RPK* genes from *Triticum urata*, seven *RPK* genes from *Triticum turgidum*, four *RPK* genes from *Aegilops tauschii*, 11 *RPK* genes from *Triticum speltoides*, four *RPK* genes from *Hordeum vulgare*, and 15 *TaRPK1* genes in *Triticum aestivum*. The number of *TaRPKs* identified in *T. aestivum* is similar to that of *RPKs* in *Triticum speltoides* (11). The identified *RPK* genes were confirmed for the conserved domains by the SMART database. A higher number of *TaRPK1* genes might be because of the large allohexaploid nature of the bread wheat genome.

The allohexaploid *T. aestivum* genome was originated due to the 3A, B, and D diploid sub genomes hybridization (Marcussen et al., 2014). Three homoeologous genes at a minimum should be for each *T. aestivum* gene, that is, one from each sub genome, also named as homoeologous genes for their homologous chromosomal localization (Sharma et al., 2016). The genome wide analysis displayed that *TaRPK1* genes along with the homoeologous genes were located mainly on chromosomes 1, 2, and 3 on A, B, and D sub-genomes, which showed that there might be no deletion of *TaRPK1* genes in the course of the acclimatization and evolution process of *T. aestivum*. The *TaRPK1* genes were found to be with maximum number on chromosome 2 and 3 (Figure 1) which was very similar to other studied crops. Crops such as *Triticum dicoccoides, Aegilops tauschii*, *Hordeum vulgare*, *Triticum speltoides,* and *Triticum turgidum* also showed the distribution of *RPKs* on chromosome 2 and 3, in addition to chromosome 5. However, the *RPKs* were distributed on chromosomes 3, 4, and 7 in rice, and in *Arabidopsis thaliana* they were on chromosomes 1 and 3*.*

The phylogenetic relationship was studied using complete TaRPK1 protein sequences, as it indicated evolutionary inference. The known homoeologous sequences were clustered closely (Figure 2), which indicated further evolutionary relationships and homology of sequences among them. The putative paralogous sequences were grouped together by those that specified similar origins. Similarity in organization and architecture of domains and motifs in clades designates functional association between these proteins. The gene structure analysis revealed intron numbers in *TaRPK1* genes that ranged from 0 to 1 and 16-18 (Figure 3A). The difference in the number of exons in *TaRPK1* was analogous to the one observed in other crops. *Triticum dicoccoides, Aegilops tauschii*, *Hordeum vulgare,* and *Triticum speltoides* exhibited one to two coding exons, and *Triticum turgidum* had one to three coding exons. *Oryza* species also exhibited one to three exons except for *Oryza rufipogon*; ORUFI04G26970 had 102 exons and Os05t0486100-01 *RPK1* exhibited 18 exons. This points toward evolutionary conservation and hence expression of genes between these species.

Prediction of protein domain configuration revealed the similarity to the previously studied RPK proteins (Cheng et al., 2009), with conserved C-terminal Ser/Thr kinase, a transmembrane domain suggesting membrane-bound features of TaRPK1 proteins and LRR domains. The LRR domains were absent in TaRPK1, TaRPK2, and TaRPK3 proteins. However, all *TaRPK1* members showed an additional LRRNT_2 (leucine-rich repeat N-terminal) domain in the N-terminal region of the amino acid (Supplemental Figure S1). In addition to sequence alignment, motif analysis also displayed the conservation of the motif at the initial N-terminal region and kinase domain with the motif that remained conserved in all of the 15 TaRPK1 protein sequences (Figure 3B). For the functional analysis of *TaRPK1* genes, the gene ontology enrichment analysis was performed (Figure 4). *In silico* prediction showed that *TaRPK1* members were involved in several processes of development through regulation of molecular functions (MFs) and biological processes (BPs), and exhibited response to environmental stresses. Several prior studies also described that through monitoring expression of genes, microRNAs respond to stress stimuli (Yan et al., 2019; Rasool et al., 2021; Rehman et al., 2022). The microRNAs are 21–24 nucleotides long endogenous non-coding RNAs that regulates development, growth, and adaptive response against abiotic stresses via monitoring target genes at posttranscriptional level or translation level of protein synthesis (Bai et al., 2017). In this study, we recognized microRNAs and their target genes in order to explore specific transcripts involved in development and growth processes and in response to different stress environments. We identified that miRNAs are majorly involved in cleavage mechanisms rather than translation inhibition (Figure 5).

The *cis*-regulatory elements identified in *TaRPK1* were mostly related to light responsiveness (Figure 6A). Other distributed *cis*-regulatory elements were related to stress factors, such as drought, cold stress, anaerobic response, wounding pathogens, and defensive elements. Functional relation of other *cis* elements was linked to plant hormones comprising auxins, abscisic, gibberellin and salicylic acid. Thus, the occurrence of various groups of *cis*-regulatory elements functioning in diverse physiological processes is suggestive of the dynamic *RPK1* gene regulation in *T. aestivum.* Synteny analysis with other ancestral *Triticum* species revealed that the *RPK1* gene family converges to a single ancestor (Figure 6B). This relationship validates that *RPKs* with analogous evolutionary status might have similar functions in plant growth and development. Homology models for 15 TaRPK1 proteins were made and evaluated with homologous templates. The TaRPK1 proteins exhibited 28%–45% identity to the template, which is a widely accepted threshold for successful modeling. The Ramachandran plots verification and validation displayed that a very higher percentage of all 15 TaRPK1 protein regions showed highly favorable regions that denote good quality protein structure prediction (Figure 7). Previous studies have shown similar 3D structure of TATrx proteins in wheat through homology modeling along with Ramachandran plot. The proteins were compared to 2iwt.1. A, 2vlt.1. A, 1fb0.1. A, 3d22.1. A, 2vm1.3. A, and 1faa.1. A templates, and the Ramachandran plot showed more than 95% of the thioredoxin amino acids lying in the most favored area (Bhurta et al., 2022). Another study in wheat has shown similar three-dimensional structure prediction of twenty-one TaEIL proteins via SWISS-MODEL along with Ramachandran plot analysis. The prediction model on the basis of templates heuristically enhanced percentage identification, alignment range, and confidence score of test sequences. The Ramachandran plot analysis confirmed 80% of residuals in the allowed area, signifying the quality of the model (Yi-Qin et al., 2020).

The gene expression in a specific tissue can be used as an information source for function identification in that tissue. Studies have revealed that *OsRPK1* overexpression altered the total architecture of roots in transgenic seedlings along with height, tillering numbers, and apical meristem of roots (Zou et al., 2014). The larger root system might result in a substantial upsurge in water and nutrient uptake. The relative expression level in different tissues of *OsRPK1* was studied, which indicated higher to lower expression in the pattern of root tips > leaf blades > roots > leaf sheath > stem (Zou et al., 2014). Alike expression pattern was also detected in *TaRPK1* genes. The heatmap generated showed significant expression of *RPK1* genes in the root tissIes in comparison to the other tissues studied (Figure 9). As gene expression profiles are always related to their function, we further investigated their expression profiles in various tissues and varieties under normal and drought stress responses. Results of real-time quantitative PCR indicated that *TaRPK1* showed higher expression levels in root tissues at seedling and heading stages under normal conditions. *TaRPK1* exhibited tissue specific expression and showed higher expression in drought stress treatment in root tissues (Figure 10). This high expression in particular organs like roots indicates their particular roles in the root development and function of that tissue. The higher expression of *TaRPK1* genes was observed in Pakistan 13 > Galaxy > Shafaq varieties, which indicates the vital role of *TaRPK1* in plant growth and development. The sequence similarity and conserved domains of these protein kinases from *Arabidopsis*, rice, and wheat combined with the evidence from *in-silico* expression analysis and RT-PCR suggest that *TaRPK1* might share analogous functions in root development and hence yield. Future functional validation of these genes will be required.

## Conclusion

We completely investigated the properties, developmental, location on chromosomes, *cis*-components, synteny, and expression profiles of *TaRPK1* members. An aggregate of 15 *TaRPK1*s were distinguished in the *T. aestivum* genome. This work can fill in as an initial phase in the complete useful portrayal of *RPK1* genes by reversible genetic methodologies. This study provides helpful assets to future investigations on the design and function of *RPK1* genes and for distinguishing and describing these genes in different species. Consequently, the outcomes might offer important data to examine the role of *TaRPK1* genes being developed and stress reactions through present-day practical genomics tools (next-generation sequencing) and genome editing, henceforth clearing the way toward genetic improvement of wheat.

## Data Availability

The original contributions presented in the study are included in the article/Supplementary Material; further inquiries can be directed to the corresponding author.
