# Supplementary material for: Genome-Wide Identification and Characterization of Receptor-Like Protein Kinase 1 (RPK1) Gene Family in Triticum aestivum Under Drought Stress
Source: Front Genet. 2022 Jul 4;13:912251. doi: 10.3389/fgene.2022.912251 (PMC9289140; doi:10.3389/fgene.2022.912251)
Supplement: Supplementary file 2 [file Table1.DOCX]

**Genome Wide Identification and Characterization of *Receptor-Like Protein Kinase 1 (RPK1)* Gene Family in *Triticum aestivum* under drought stress**

**Amna Abdul Rahim^1,2^, Muhammad Uzair^2^, Nazia Rehman^2^, Obaid Ur Rehman^2^, Nageen Zahra^2^, and Muhammad Ramzan Khan^1,2,*^**

^1^National Centre for Bioinformatics (NCB), Quaid-e-Azam University, Islamabad 45320, Pakistan. [amnaraheem786@gmail.com](mailto:amnaraheem786@gmail.com) (AR)

^2^National Institute for Genomics and Advanced Biotechnology, National Agricultural Research Centre, Park Road, Islamabad 45500, Pakistan

[uzairbreeder@gmail.com](mailto:uzairbreeder@gmail.com) (MU); [naziarehman9@yahoo.com](mailto:naziarehman9@yahoo.com) (NR); [obaid.sheikh@hotmail.com](mailto:obaid.sheikh@hotmail.com) (OR); [nageenzahra@hotmail.com](mailto:nageenzahra@hotmail.com) (NZ)

^*^Correspondence: [mrkhan@parc.gov.pk](mailto:mrkhan@parc.gov.pk) (MRK)


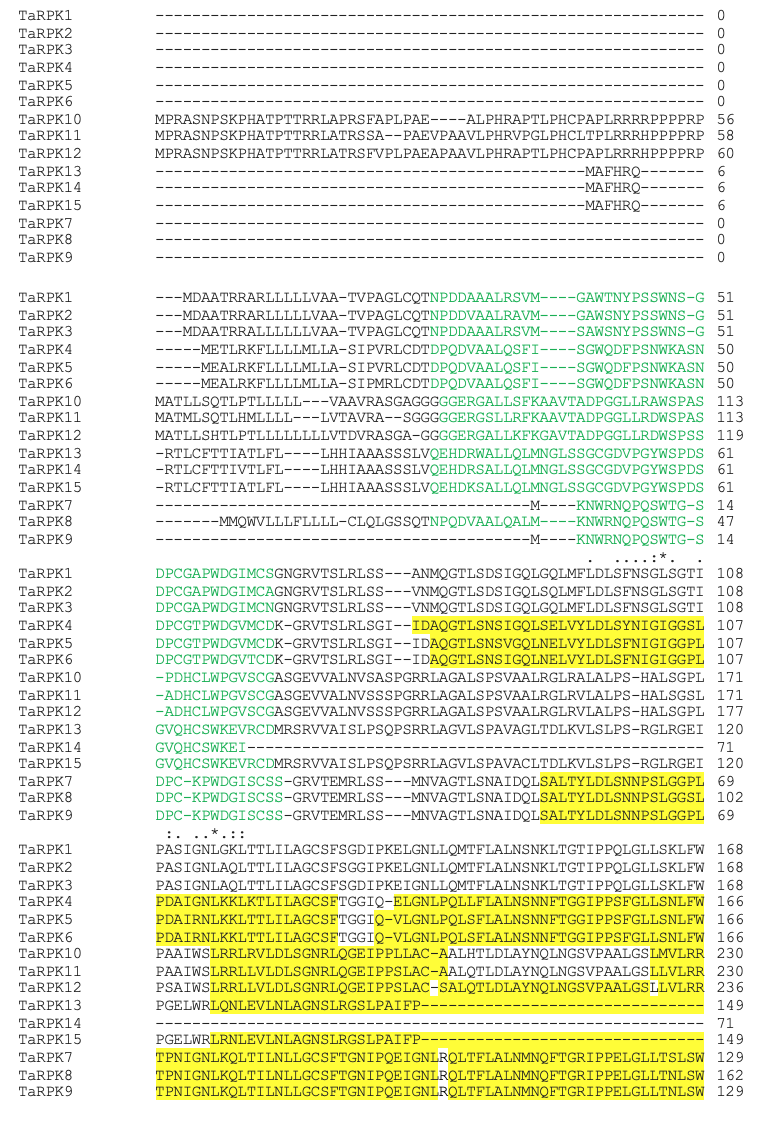


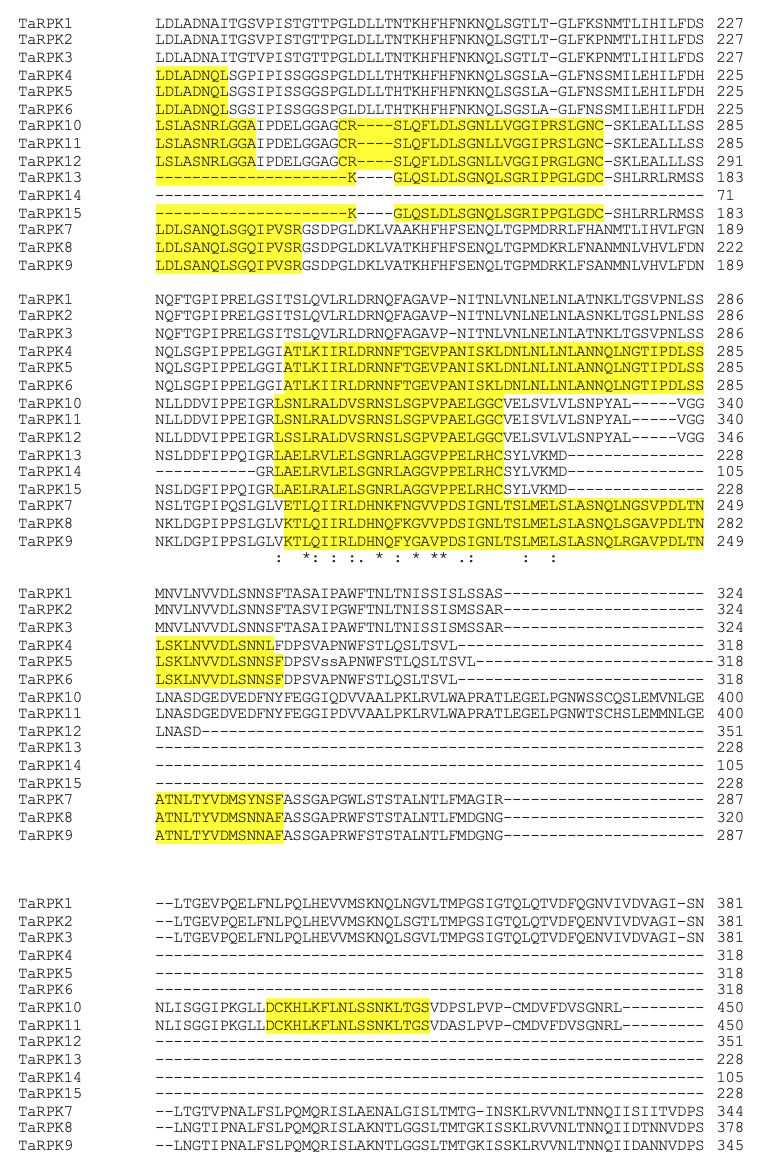


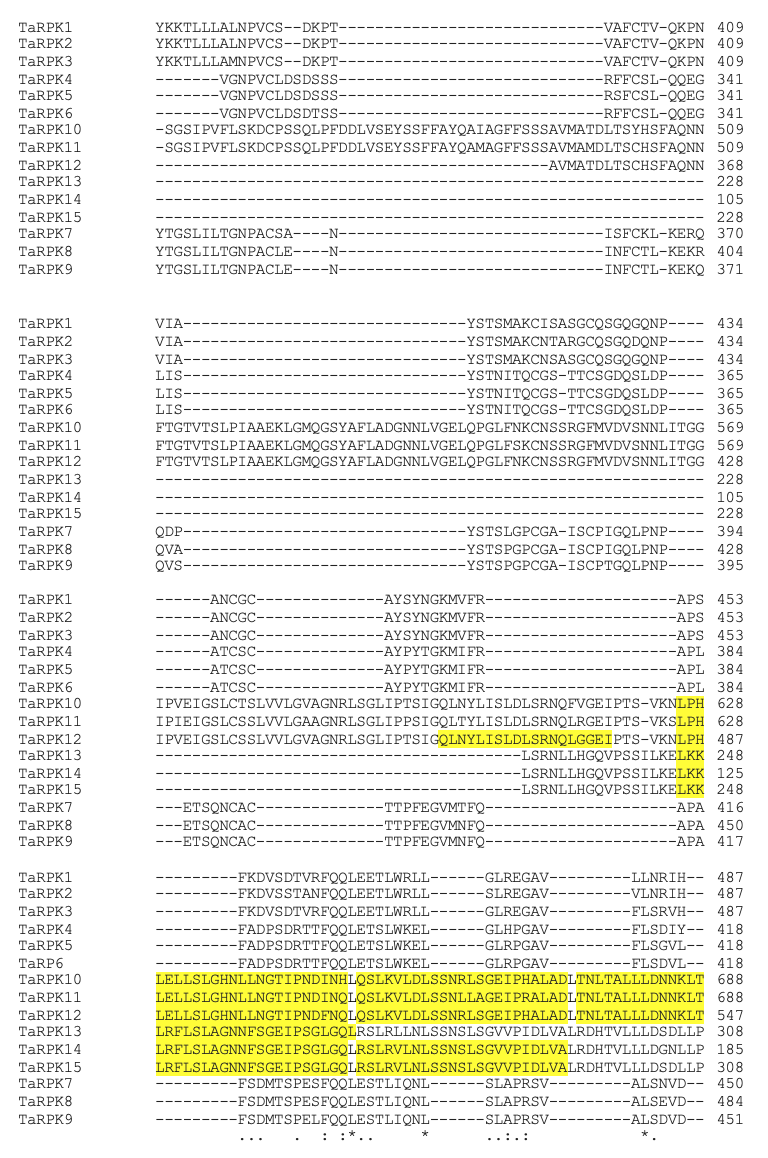


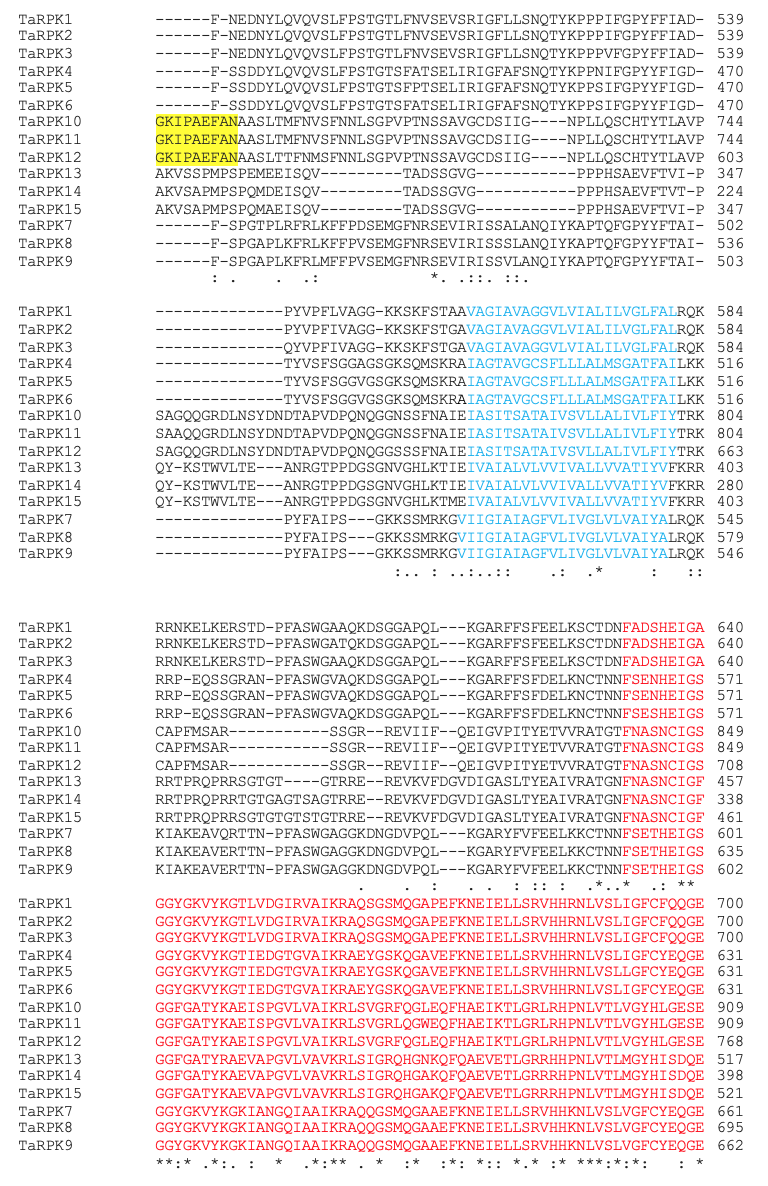


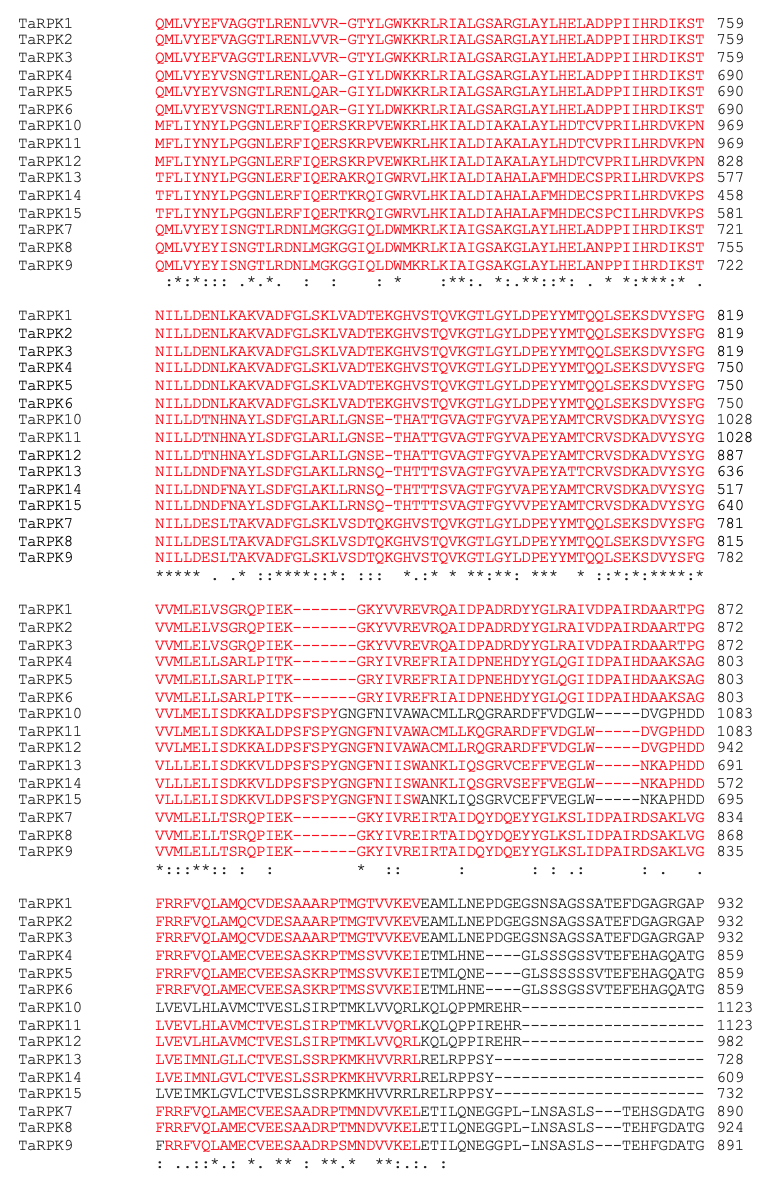


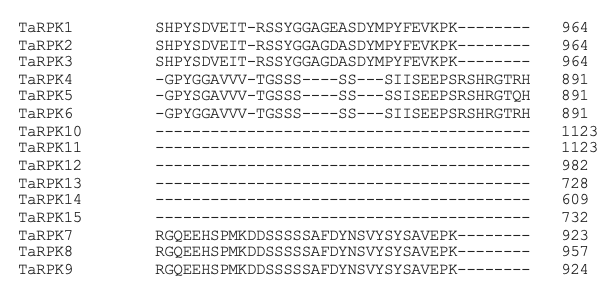


* = identical amino acid residues in all sequences

: = highly conserved amino acids

. = different but somewhat similar amino acids

blank = dissimilar amino acids or gaps

**Supplemental Figure S1.** Sequence alignment of TaRPK1 proteins depicting structural predictions. Green color indicates LRRNT_2 Domain (Leucine Rich Repeat N-Terminal Domain), yellow color indicates LRR (Leucine Rich Repeats), blue color denotes TM (Transmembrane domain) and red color indicate STKc (Kinase domain).

**
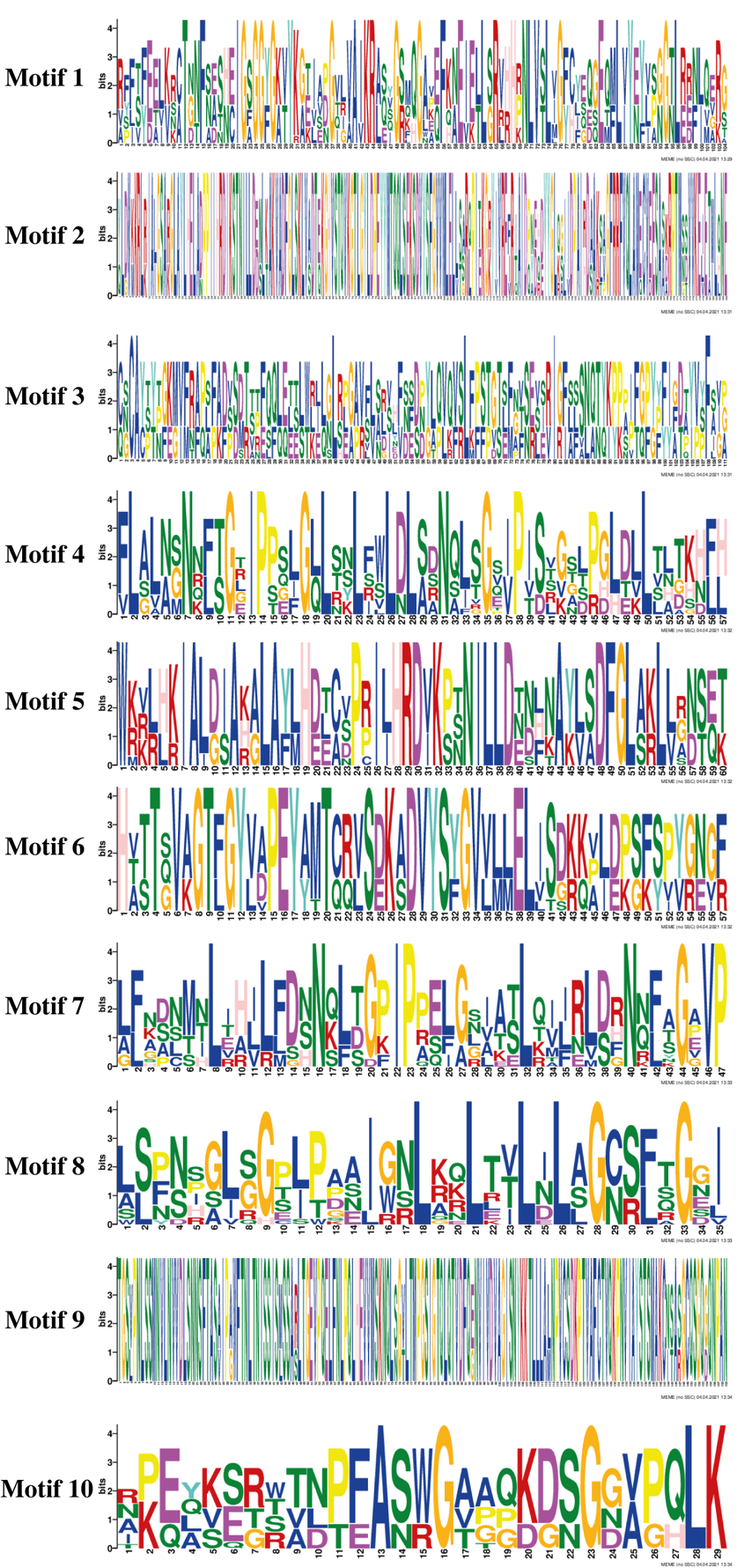
**

**Supplemental Figure S2.** Sequence logos with in the *TaRPK*.


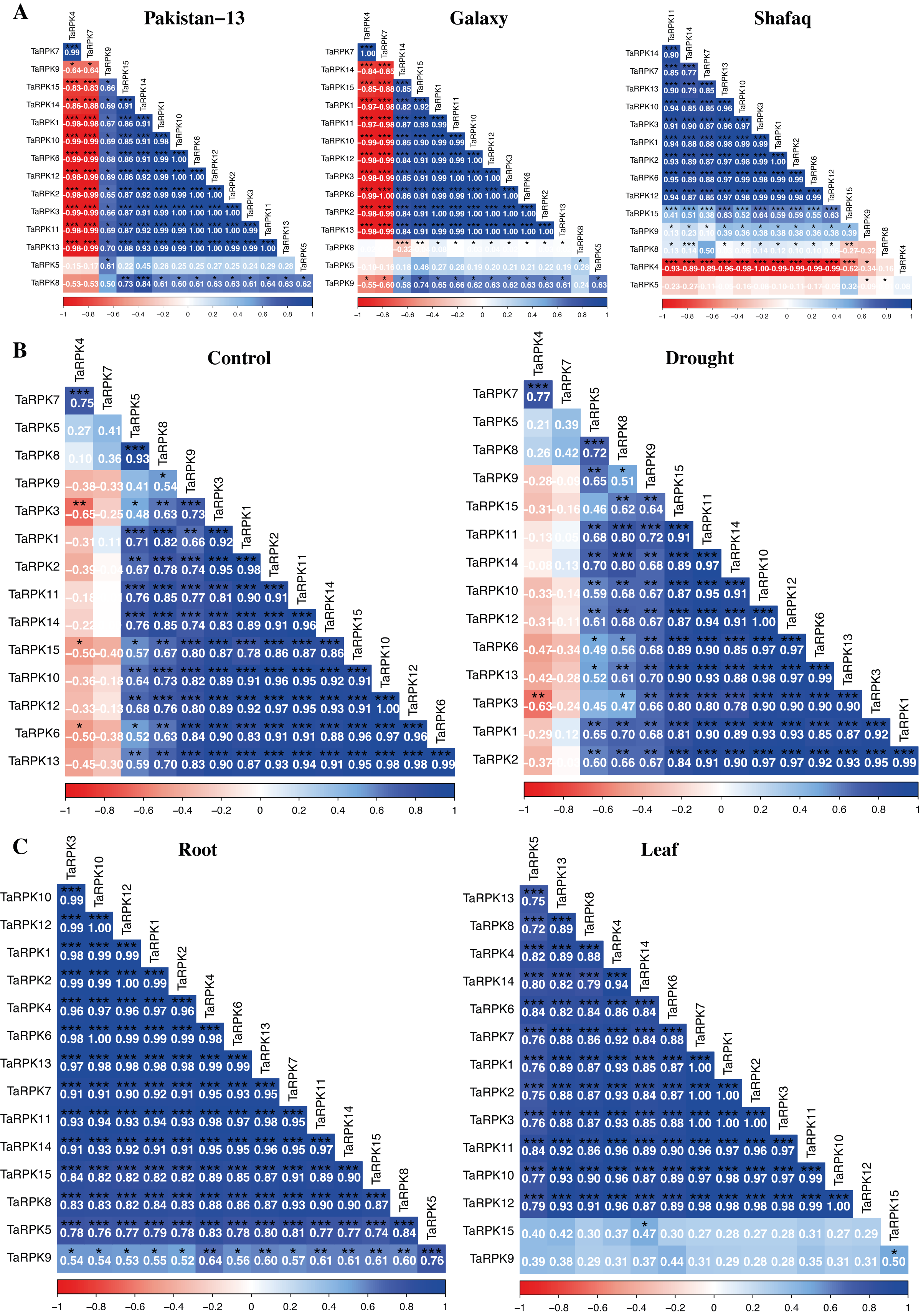


**Supplemental Figure S3.** Co-expression analysis among *TaRPK1* members on the bases of verities (A), treatments (B), and tissues (C).


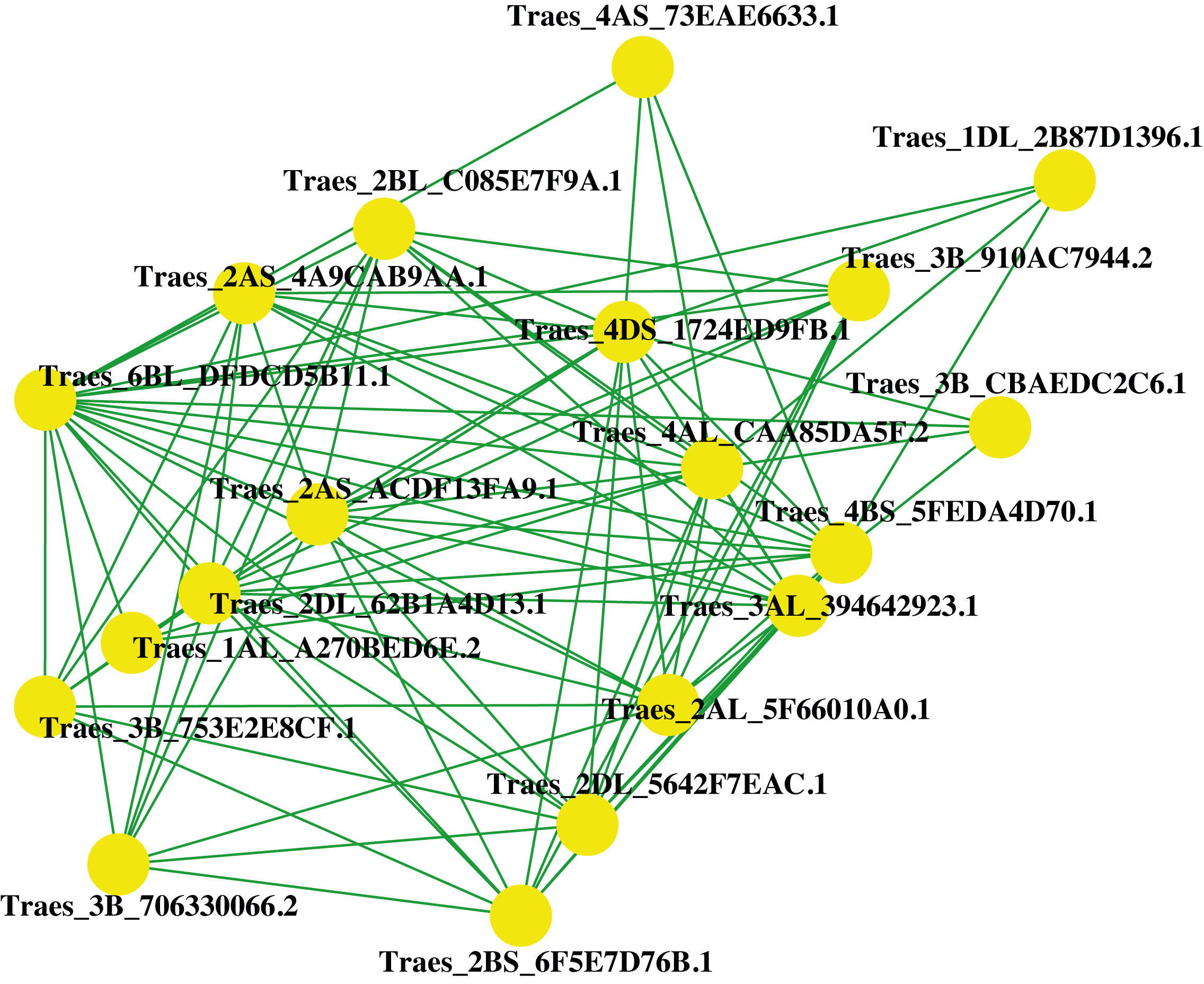


**Supplemental Figure S4.** Network interaction analysis among TaRPK1 gene family.
